# Awareness and knowledge regarding of cervical cancer, Pap smear screening and human papillomavirus infection in Gabonese women

**DOI:** 10.1186/s12905-015-0193-2

**Published:** 2015-04-19

**Authors:** Samira Zoa Assoumou, Barthelemy Mabika Mabika, Angelique Ndjoyi Mbiguino, Mustapha Mouallif, Abdelkim Khattabi, My Mustapha Ennaji

**Affiliations:** Laboratoire de Virologie, Microbiologie et Qualité/Eco toxicologie et Biodiversité, Faculté des Sciences et Techniques de Mohammedia, Université Hassan II- Casablanca, Casablanca, Maroc; Laboratoire d’Agroalimentaire et Santé, Département de biologie, Faculté des Sciences et Techniques, Université Hassan I, Settat, Maroc; Département d’Anatomie et de Cytologie pathologiques, Faculté de Médecine et des Sciences de la Santé, Université des Sciences de la Santé, Libreville, Gabon; Laboratoire de référence MST/Sida, Laboratoire de référence rougeole, rubéole et fièvre jaune, Département de Bactériologie et Virologie, Faculté de Médecine et des Sciences de la Santé, Université des Sciences de la Santé, Libreville, Gabon; Laboratory of Experimental Pathology, University of Liege, Liege, Belgium

**Keywords:** Cervical cancer, Pap smear test, HPV, Knowledge, Gabonese women

## Abstract

**Background:**

Cervical cancer is the commonest cancer and the leading cause of cancer mortality in women in Gabon. The age-standardized incidence of cervical cancer is 19.9 per 100 000 women and the mortality rate is 8.4 per 100 000. Various international studies have identified the lack of awareness and knowledge about cervical cancer as barriers to use preventive methods. This article assesses the awareness and knowledge about cervical cancer, Pap smear testing and its use and HPV among women living in Libreville, Gabon.

**Methods:**

This study was conducted in October 2014 in Libreville. A total of 452 women aged 16 years and above were recruited from different town locations. Logistic regression analysis was used to identify the effect of demographic characteristics on the level of knowledge about cervical cancer, Pap smear testing and HPV. Odds ratio and 95% confidence intervals were used to identify the strength of association. Associations were considered statistically significant at p < 0.05.

**Results:**

Of all the women interviewed, 91.6% (414/452) had heard about cervical cancer and only 27.9% (126/452) had heard of Pap smear test. Of these 126 women, only 65.1% (82/126) had done cervical cancer screening and 68.3% (56/82) on the suggestion of a doctor. The most common reason for not undergoing Pap smear testing was neglect (50%, 22/44) followed by lack of financial resources (13.6%, 6/44), fear of discovering a serious disease (13.6%, 6/44) and deeming it unimportant (13.6%, 6/44). Only 8% (40/452) of the participants had heard about HPV and their knowledge of HPV was fair. There is a very poor level of knowledge about cervical cancer among Gabonese women.

**Conclusion:**

This study demonstrates a very low level of knowledge about cervical cancer, Pap smear testing and HPV in a sample of Gabonese women. There is a critical need for Gabonese women to be informed about cervical cancer and the Pap smear test to improve the use of this preventive method. The implication of health staff and Gabonese media should be included as a centerpiece in the effort to inform the population in order to reduce the burden of cervical cancer in Gabon and save women lives.

## Background

Cervical cancer is the fourth most common cancer among women worldwide, with an estimated 528,000 new cases and 266,000 deaths in 2012 [1]. Human papillomavirus (HPV) is thought to be the most common sexually transmitted infection worldwide and more than 75% of sexually active adults have had HPV infection in their lifetime [2]. The correlation between the presence of this virus and the development of precancerous lesions that may lead to cervical cancer is clearly established.

Cervical cancer is one of the few preventable human cancers, its prevention is based on the early diagnosis of precancerous lesions whose treatment generally makes the development of cancer almost impossible [3]. However, in developing countries, cervical cancer remains the most common cause of cancer-related deaths among women [4]. In developed countries, the incidence and mortality of this cancer have decreased significantly because of the efforts made to detect precancerous lesions early [5,6].

Despite the high prevalence of cervical cancer, many studies have shown that women’s knowledge about HPV, cervical cancer and cervical screening is very low [7-10]. Moreover, the uptake and success of cervical cancer screening is determined by women’s knowledge and awareness of cervical cancer [10].

In Gabon, a country in middle Africa, cervical cancer is considered a major public health problem and is the most commonly occurring cancer among Gabonese women [11]. According to the 2012 International Agency for Research on Cancer (IARC) report, the age-adjusted incidence rate of cervical cancer in Gabon is 19.9 per 100,000 (118 new cases/year) and the mortality rate from cervical cancer is 8.4 per 100,000 (48 deaths/year)[1].

No data are currently available regarding awareness and knowledge of cervical cancer, Pap smear screening and HPV among Gabonese women. Thus, this study aimed to assess the knowledge and awareness of cervical cancer, Pap smear testing and HPV in Gabonese women and reveal which sources of information are commonly used in their health education.

## Methods

### Participant recruitment

A questionnaire-based survey, using face-to-face interviews, was carried out in October 2014 in Libreville, the capital of Gabon. Women aged 16 years and above were approached in different public sites of the town (market, mall, public garden, church etc.…), thus increasing the chance of covering women from different social groups. A total of 452 women agreed to voluntarily participate in the study. The women were interviewed by two trained researchers who had extensive knowledge of cervical cancer, screening and HPV. The interviews were conducted in French, the national language of the country.

### Questionnaire data and process

A questionnaire based on a literature review and a previously published questionnaire [12] was designed in English. The questionnaire was translated into French and then translated back into English for quality assurance. The items on the questionnaire were divided into four sections: (i) demographic characteristics of the participants, and awareness, knowledge, and information sources of (ii) cervical cancer, (iii) Pap smear testing and (iv) HPV.

Information on age, marital status, occupation, number of children, level of education and monthly income was collected. Monthly income (Central African CFA Franc) was categorized as “low” (<200 000) “medium” (200 100–400 000) and “high” (>400 000).

During the interview, if the participant had never heard about cervical cancer, she was asked if she had ever heard about Pap smear testing. If the answer was again no, she was asked if she had ever heard about Human Papillomavirus/HPV. If this answer was also no, the questionnaire was finished for this participant, to eliminate answers due to guessing.

Two knowledge scores were created: one for the entire population of the study and one to measure the knowledge level about HPV for women who had heard about HPV. The participants received 1 point for correct answers and 0 for either wrong answers or when the answer was “I don’t know”. Questions regarding specific knowledge about cervical cancer, Pap smear testing and HPV were considered to create the overall knowledge score. The knowledge questions were grouped and the mean score was computed to determine the overall knowledge of participants. The participants who scored average or above were considered as “knowledgeable” and the others as “not knowledgeable”.

For participants who answered questions about HPV (on the basis that they had indicated that they had heard of HPV), three levels of knowledge were defined: a low level of knowledge (0–2 correct answers), a fair level of knowledge (3–4 correct answers) and an excellent level of knowledge (5–6 correct answers).

The questionnaire was piloted on a sample of 10 female volunteers who were not included in the study. The interviews lasted 7 minutes on average. At the end of the questionnaire, an information session about the disease was given to the participant when they answered ‘yes’ to the last question: “Do you think you need information about this disease?”

### Ethical considerations

Ethical approval (number: 00287/MS/SG) was obtained from the “Ministère de la Santé de Libreville”. All participants gave written consent (for participants 17 years and below, written consent was obtained in addition to parental consent). Confidentiality was ensured through the use of code numbers rather than participants’ names.

### Data analysis

Statistical analysis was performed using Epi info6 software (www.epivf.fr). Logistic regression analysis was used to identify the effect of demographic characteristics on the level of knowledge about cervical cancer, Pap smear testing and HPV. Odds ratio and 95% confidence intervals were used to identify the strength of association. Associations were considered statistically significant at p < 0.05.

## Results

### Demographic characteristics of the participants

The mean age of participants was 30.4 years (range 16–65 years). Of the interviewed women, 50.4% (228/452) reported never being married, 63.7% (288/452) had a university level of education (not necessarily completing university) and 51.6% (233/452) were employed. The majority of the women were of low income (69.1%, 312/452). Demographic characteristics are shown in Table 1.

**Table 1:** **Characteristics of the study participants**

**Characteristics**	**N**
**Age**	
<30	268 (59.3)
30-39	104 (23.0)
40-49	56 (12.4)
≥50	24 (5.3)
**Marital status**	
Never married	228 (50.4)
Ever married	224 (49.6)
**Occupation**	
Student	184 (40.7)
Unemployed/housewife/retired	35 (7.7)
Employed	233 (51.6)
**Education**	
No school	10 (2.2)
Primary school	12 (2.7)
Secondary school	142 (31.4)
University	288 (63.7)
**Monthly household income**	
Low	312 (69.1)
Medium	72 (15.9)
High	68 (15.0)

**N** = Number of respondents.

Percentage are shown in parentheses.

### Awareness and knowledge about cervical cancer

Of the 452 participating women, 91.6% (414/452) had heard of cervical cancer. Among these women, 62.3% (258/414) felt moderately/extremely concerned about cervical cancer (Table 2). Only 22.7% (94/414) of women reported knowing the causes of cervical cancer. The most frequently cited causes of cervical cancer were abortion and sexually transmitted infection (including HPV) respectively in 29.8% (28/94) and 28.7% (27/94). The less frequently cited causes of cervical cancer were early pregnancy and high parity (2.1%, 2/94 each).When asked about the possibility of developing cervical cancer one day, 40.4% (38/94) of women believed they had no risk. Participating women acquired information about cervical cancer mostly from either foreign media or medical workers: 28.5% (118/414) and 27.1% (112/414) respectively. Results regarding cervical cancer, Pap smear testing and HPV information sources are presented in Table 2.

**Table 2:** **Participants’ awareness and knowledge of cervical cancer, and information source about cervical cancer, Pap smear testing and HPV**

**Questions**	**N**
**Have you ever heard of cervical cancer?**	
No	38.0 (8.4)
Yes	414.0 (91.6)
**Are you concerned about having/developing cervical cancer?** ^**1**^	
No	90.0 (21.7)
A little	66.0 (15.9)
Moderately	96.0 (23.2)
Extremely	162.0 (39.1)
**Do you know the risk factors/causes of cervical cancer?**	
No	320.0 (77.3)
Yes	94.0 (22.7)
Risk factors for cervical cancer cited by the respondents*	
Abortion	28.0 (29.8)
Sexually transmitted infection	27.0 (28.7)
Smoke	21.0 (22.3)
Multiple partners	19.0 (20.2)
Insert products/fingers into the vagina	17.0 (18.1)
Sex at an early age	14.0 (14.9)
Lack of hygiene	10.0 (10.6)
Alcohol/Drug	7.0 (7.5)
Excessive sex	4.0 (4.3)
Heredity	3.0 (3.2)
High parity	2.0 (2.1)
Early pregnancy	2.0 (2.1)
**Self-perceived chance of developing cervical cancer** ^**2**^ **?**	
Non existent	38.0 (40.4)
Very low	20.0 (21.3)
low	12.0 (12.8)
moderate	14.0 (14.9)
High	10.0 (10.6)
**Information source about cervical cancer?**	
Foreign media	118.0 (28.5)
Gabonese media	112.0 (27.1)
Medical staff	108.0 (26.0)
Family/friends	76.0 (18.4)
**Information source about Pap smear test?**	
Medical staff	66.0 (52.4)
Foreign media	30.0 (23.8)
Gabonese media	16.0 (12.7)
Family/friends	14.0 (11.1)
**Information source about HPV?**	
Foreign media	26.0 (65.0)
Medical staff	14.0 (35.0)
Gabonese media	0.0 (0.0)
Family/friends	0.0 (0.0)

**N** = Number of respondents.

^**1**^Only for women who have heard about cervical cancer.

^**2**^Only for women who cited at least one risk factors of cervical cancer.

*This was an open question; the total percentage can exceed 100% because of multiple answers.

Percentage are shown in parentheses.

### Awareness, knowledge and attitude toward Pap smear testing

Only 27.9% (126/452) of the study participants had heard about cervical cancer prevention through screening. Among these, 65.1% (82/126) had had a Pap smear previously. The principal motivation to undergo Pap smear testing as cited by these women was “the demand of their doctor” (68.3%, 56/82). Some women reported being embarrassed about the test (61%, 50/82) and had experienced pain during screening (34.1%, 28/82). The main reason given by women who had never been screened for cervical cancer but had heard about it was neglect (50%, 22/44). Table 3 summarizes the knowledge and attitudes about cervical screening in the study population.

**Table 3:** **Knowledge and attitudes toward the Pap smear test**

**Questions**	**N**
**Have you ever heard about Pap smear test?**	
No	326 (72.1)
Yes	126 (27.9)
We use pap smear test*:	
To prevent cervical cancer	108 (85.7)
To prevent infection in genital tract of women	34 (27.0)
To prevent sexual transmitted infection in general	6 (4.8)
**Undergone a pap smear test:**	
No	44 (34.9)
Yes	82 (65.1)
**How many times?**	
At one time	40 (48.8)
2-3 times	34 (41.5)
>3times	8 (9.8)
**Motivation to undergone a pap smear test:**	
On demand of doctor	56 (68.3)
By yourself	12 (14.6)
Both	14 (17.1)
**Feel pain during Pap smear test?**	
Yes	28 (34.1)
No	54 (65.9)
**Feel embarrassed?**	
Yes	50 (61.0)
No	32 (39.0)
**Normal frequency for doing pap smear test:**	
Every year	64 (78.1)
Every 2-3years	18 (21.9)
**Reasons for not making pap smear test:**	
negligence	22 (50.0)
I don’t have the financial resources	6 (13.6)
I think it is not important	6 (13.6)
I am afraid to discover a grave disease	6 (13.6)
I am afraid of pain	4 (9.1)

**N** = Number of respondents.

Percentage are shown in parentheses.

*Can exceed 100% because of multiple answers.

### Awareness and knowledge of HPV

Among all the participants, only 8.8% (40/452) had heard of HPV. The majority of respondents reported that HPV is the principal risk factor of cervical cancer (65%, 26/40) and was acquired sexually (70%, 28/40) (Table 4). The answers to the HPV questions were used to create a knowledge score. Analysis revealed that 35% (14/40) of women had a low level of knowledge (0–2 correct answers), 60% (24/40) had a fair level of knowledge (3–4 correct answers), and only 5% (2/40) had a high level of knowledge (5–6 correct answers).

**Table 4:** Participants’ knowledge of HPV*

**Questions**	**Participant’s correct answer**
HPV can cause urinary infection?	8(20%) false
HPV is the principal risk factor of cervical cancer?	26 (65%) yes
HPV can cause genital warts?	20 (50%) yes
HPV infect only women?	16 (40%) false
HPV is a sexually transmitted infection?	28 (70%) yes
Condom gives total protection against HPV?	20 (50% )false

*Only for the 40 women who have heard about HPV.

### Factor related to level of knowledge

Knowledge of cervical cancer, Pap smear testing and HPV was related to the socio-demographic characteristics of the participants. The women generally demonstrated a poor level of knowledge (93.6%, 423/452). Older participants, those who had ever been married and those with medium to high monthly incomes were more likely to have a good knowledge (Table 5).

**Table 5:** **Socio-demographic characteristics that correlate to the level of knowledge about cervical cancer among Gabonese women**

**Knowledge of cervical cancer, pap smear test and HPV (N = 452)**			
**Variables**	**Yes, N = 29 (6.4%)**	**No, N = 423 (93.6%)**	**OR (95% IC)**
**Age**			
<30	7 (2.6%)	261 (97.4%)	7.5 (2.0-27.6)*
30-39	8 (7.7%)	96 (92.3%)	2.4 (0.7-8.7)
40-49	10 (17.9%)	46 (82.1%)	0.9 (0.3-3.3)
≥50	4 (6.7%)	20 (83.3%)	1
**Occupation**			
Student	4 (2.2%)	180 (97.8%)	2.7 (0.5-15.5)
Not employed	2 (5.7%)	33 (94.3%)	1
Employed	23 (9.9%)	210 (90.1%)	0.5 (0.1-2.5)
**Educational level**			
No school	0 (0.0)	10 (100%)	1
Primary education	0 (0.0)	12 (100%)	1.2 (0.02-65.3)
Secondary education	0 (0.0)	142 (100%)	13.6 (0.2-718.9)
University	29 (10.1%)	259 (89.9%)	0.4 (0.02-7.3)
**Marital status**			
Never married	9 (3.9%)	219 (96.1%)	1
Ever married	20 (8.9%)	204 (91.1%)	0.4 (0.2-0.9)*
**Montly household income**			
Low	6 (1.9%)	306 (98.1%)	1
Medium	6 (8.3%)	66 (91.7%)	0.2 (0.1-0.7)*
High	17 (25%)	51 (75%)	0.1 (0.02-0.2)*
**Parity**			
Nulliparous	6 (3.7%)	155 (96.3%)	1
At least one child	23 (7.9%)	268 (92.1%)	0.5(0.2-1.1)

*Statistically significant.

Average knowledge score = 8.5.

## Discussion

The current study is the first to explore the level of knowledge and awareness of cervical cancer, Pap smear testing and HPV in Gabonese women. The findings reflect a poor level of knowledge. Such findings have been reported by a number of previous studies in other African communities [7,8,13-15] and was not a surprising cause in this part of the world effort to inform population about cervical cancer is still lacking. Different socio-demographic variables such as higher income, older age and being married were found to be predictors of a good knowledge level of cervical cancer. The findings from the current study are consistent with several other African studies [7,8,16]. However, in the current study, the level of knowledge was not associated with the participants’ occupation or educational level, probably because of the high proportion of students (including postgraduate students) in the interviewed women and the type of courses studied by these women. In Gabon there is no information program about cervical cancer available to students other than those enrolled in the health academic program. These findings highlight the need to inform students about cervical cancer by introducing informative programs into the education system.

In this study, 91.6% (414/452) of women had heard about cervical cancer but only 22.7% (94/414) reported knowing the causes of cervical cancer. However, there were common knowledge gaps concerning the risk factors of cervical cancer. The risk factors that were most frequently cited included abortion, sexually transmitted infection, smoking, multiple sexual partners, inserting products/fingers into the vagina, sex at an early age and lack of hygiene. In African societies, incorrect risk factors for cervical cancer such as abortion, lack of hygiene and the insertion of products/fingers into the vagina are commonly cited by women [8,13]. This suggests that accurate information about cervical cancer and its causes needs to be available to women through mass campaigns that also dispel false traditional beliefs about cervical cancer. The others risk factors commonly cited by women were related to sexual comportment and were identified in the literature as risk factors for cervical cancer [17]. Smoking was cited as a risk factor for cervical cancer by 22.3% (21/94) of participants in the current study. In many African studies, the risk posed by smoking is unknown or cited by a small proportion of women [8,13,16,18]. Findings from the current study suggest that Gabonese women predominantly associate cervical cancer with sexual behavior. High parity, early age at first pregnancy, heredity, excessive sex and alcohol/drug were less frequently cited by the women. High parity and early age at first pregnancy have been described in the literature as risk factors for cervical cancer [17,19]; however, the role of heredity and alcohol/drug use remains unclear [17,20]. A high proportion of women (62.3%) reported that they were moderately/extremely worried about having/developing cervical cancer. However, 40.4% of women believed that they were at no risk of developing cervical cancer. In this scenario, it is important to correct the false perceptions about cervical cancer among Gabonese women.

In Gabon, although cervical cancer has been selected as a priority disease, a national organized cervical cancer screening program is still lacking. Individual opportunistic screening is practiced and some organizations politically affiliated offer rare screening campaigns. The current study reveals that a little part of the respondents (126/452; 27.9%) had heard about Pap smear screening and 65.1% (82/126) of these women had already had a Pap smear test. However, the principal reason that these women were tested was the recommendation of their doctor, which classifies their screening as an opportunistic. In a study among Hispanic women, Austin et al. [21] reported that the recommendation of a doctor is a strong motivation to be screened for cervical cancer. This finding has also been reported among Chinese-Australian women by Kwok et al.[22]. In Africa, Obi et al. [23] reported the same result among Nigerian women. These findings highlight the importance of medical workers in informing women about cervical cancer and screening. However, the majority of screened women (61.0%, 50/82) reported being embarrassed during Pap smear testing. This feeling has been identified as an obstacle for having repeat Pap smear tests in studies conducted among Brazilian [12], Hispanic [24] and Qatari [25] women and should be taken into account to increase the compliance of women in the practice for cervical cancer screening.

In the present study, the major barrier cited by women in use for cervical cancer screening was negligence; fear of discovering a serious disease was also cited by the interviewed women. These barriers to screening were mentioned in previous studies [7,26]. Negligence may suggest the need for an aggressive information campaign about this disease. However, fear reflects a poor understanding of the natural history of cervical cancer and of the principle behind cervical cancer screening. Moreover, this suggests that the acceptability of cervical screening could be high if women were simply informed.

Several studies have reported that knowledge of HPV was low in developing countries [27-30] compared with some developed countries [31,32]. This discrepancy is due to the effort to inform the population about HPV and vaccination. The results of the current study are concordant with the existing literature on HPV knowledge. Indeed, only 8.8% (40/452) of the participants had heard about HPV. In contrast, a study conducted in Australia, where a school-based National HPV Vaccination and information Program about HPV is available, found that the majority (88.9%) of women had heard of HPV [33]. In Gabon, despite the availability of the HPV vaccines, they are not promoted by the authorities. These findings emphasize the need to improve understanding of the link between HPV and cervical cancer in Gabonese women.

Some limitations of the present study should be mentioned. The high proportion of women educated to university level (postgraduate student) is not representative of the general Gabonese population. The inclusion method is likely the main reason for this bias because the study population mainly included women who were not at work during the recruitment times and were present in the public sites. A sample size was not calculated because of the descriptive nature of this study and the unavailability of previous information about the topic. However, this is the first exploratory study of women’s knowledge about cervical cancer, Pap smear testing and HPV in Gabon and it reveals the need to inform Gabonese women about cervical cancer in Gabon.

## Conclusion

Our study highlights the lack of knowledge about cervical cancer in Gabonese women. There is a real necessity to inform Gabonese women about cervical cancer screening. Education campaigns involving the local media may be a good approach to inform Gabonese women.
